# Eg5 orchestrates porcine oocyte maturational progression by maintaining meiotic organelle arrangement

**DOI:** 10.1186/s13008-018-0037-1

**Published:** 2018-05-24

**Authors:** Yan Xie, Minghui Cheng, Shan Lu, Qilong Yuan, Dongyu Yang, Ying Chen, Chen Pan, Yurong Qiu, Bo Xiong

**Affiliations:** 1 Laboratory Medicine Center, Nanfang Hospital, Southern Medical University, Guangzhou, 510515 China; 2 Department of Reproductive Medicine, The Second Affiliated Hospital of Guangzhou University of Chinese Medicine, Guangzhou, 510120 China; 3 College of Animal Science and Technology, Nanjing Agricultural University, Nanjing, 210095 China

**Keywords:** Oocyte meiotic maturation, Spindle assembly, Actin dynamics, Mitochondrion integrity, Cortical granule

## Abstract

**Background:**

Kinesin superfamily proteins are microtubule-based molecular motors essential for the intracellular transport of various cargos, including organelles, proteins, and RNAs. However, their exact roles during mammalian oocyte meiosis have not been fully clarified.

**Results:**

Herein, we investigated the critical events during porcine oocyte meiotic maturation with the treatment of Eg5-specific inhibitor monastrol. We found that Eg5 inhibition resulted in oocyte meiotic failure by displaying the poor expansion of cumulus cells and reduced rate of polar body extrusion. In the meantime, the spindle assembly and chromosome alignment were compromised, accompanied by the decreased level of acetylated α-tubulin, indicative of less stable microtubules. Impaired actin dynamics and mitochondria integrity were also observed in Eg5-inhibited oocytes. Additionally, inhibition of Eg5 caused the abnormal distribution of cortical granules and ovastacin, a cortical granule component, potentially leading to the fertilization failure.

**Conclusions:**

Our findings reveal that Eg5 possesses an important function in porcine oocyte meiotic progression by regulating the organelle dynamics and arrangement.

## Background

To generate fertilizable female gametes, mammalian oocytes must undergo well-regulated meiotic maturation of both nucleus and cytoplasm, including resumption of meiosis, proper organelle assembly and arrangement, as well as first polar body extrusion [1]. Any errors during this process will lead to the failed meiotic progression. For example, the spindle assembly checkpoint (SAC) guarantees proper cell cycle progression. Disorganized spindle apparatus would cause the activation of SAC which in turn induces the meiotic arrest. However, if the SAC activity is silenced, aneuploid eggs will be produced, resulting in early embryonic lethality, abortion or birth defects [2]. In addition, the integrity of mitochondria and cortical granules are regarded as the critical sign of cytoplasmic maturation of oocytes [3–5]. Mitochondria provide the energy for oocyte development and cortical granules function for block to polyspermy [6, 7]. Both defects will disrupt oocyte meiotic maturation.

The kinesin is a microtubule-based motor protein family consists of 14 distinct subclasses and more than 40 proteins in eukaryotic cells [8]. A large number of these proteins, or their orthologues, are involved in various vesicle transport and intracellular trafficking events and have been shown to participate in the fundamental biological processes in both the mitosis and the meiosis such as transport of vesicles and macromolecules, microtubule dynamics, spindle formation, mitochondria translocation, chromosome separation, cytokinesis and cell cycle progression [9]. The majority of kinesins comprise two domains: an ATP hydrolysis domain that allows it to traverse along the microtubule filaments, and a tail domain that is able to bind to structures and/or cargos [8]. Most kinesins walk towards the plus end of a microtubule, which, in most cells, entails transporting cargo such as protein and membrane components from the center of the cell towards the periphery, which is known as anterograde transport [10].

Eg5 (KSP or KIF11), a member of the widely conserved kinesin-5 family, is involved in separation of centrosomes as well as bipolar spindle formation and maintenance during mitosis in vertebrates [11–15]. As reported, homotetrameric Eg5 motors possess the capacity to slide antiparallel microtubules apart in the process of spindle formation [16] and neurogenesis [17, 18]. By acting as the dominant microtubule sliding factor, Eg5 also limits the maximum speed of microtubule sliding in both spindle assembly [19] and midzone elongation [20, 21]. Furthermore, Eg5 has been suggested to play a part in tumorigenesis. Eg5 has been found to be overexpressed in breast cancer and may be served as a potential prognostic marker [22]. In meiosis, Eg5-mediated microtubule sliding drives poleward microtubule flux in spindle assembly in Xenopus egg extracts [23, 24]. In addition, Eg5 exposure increased the incidence of aneuploidy as a result of premature sister chromatid separation in mouse oocytes [25].

In the present study, we used porcine oocytes as a research model to investigate the function of Eg5 during oocyte meiotic maturation because they are developmentally and physiologically more similar to the humans [26, 27]. Our findings show that Eg5 participates in porcine oocyte meiotic progression by regulating the cytoskeleton dynamics including microtubule stability, spindle assembly, chromosome alignment and actin polymerization. We also find that Eg5 mediates the dynamic distribution of cortical granules and ovastacin in unfertilized eggs to ensure the successful fertilization.

## Methods

### Antibodies

Mouse monoclonal anti-α-tubulin FITC antibody, anti-phalloidin-FITC antibody, anti-acetyl-α-tubulin (Lys-40) antibody and LCA-FITC were purchased from Sigma (St. Louis, MO, USA); Rabbit polyclonal anti-human ovastacin antibody was obtained from Dr. Jurrien Dean. FITC-conjugated goat anti-mouse IgG (H + L) and TRITC-conjugated goat anti-mouse IgG (H + L) were purchased from Zhongshan Golden Bridge Biotechnology Co., LTD (Beijing, China).

### Inhibitor treatment

Monastrol (Calbiochem, San Diego, CA, USA) was dissolved in DMSO and diluted into a final concentration of 10, 50 and 100 μM, respectively, with maturation medium, with the final concentration of the solvent not more than 0.1% of the culture medium.

### Porcine oocyte collection and in vitro maturation

Porcine ovaries were obtained from the scale slaughterhouses of Tianhuan Meat Enterprises Co. (Nanjing, China). After 10 h rest from transport, 6-month-old pigs were stunned to unconsciousness via electronarcosis with the electrical pulse at 85 V, 1A for 3 s, and euthanasia were applied within 30 s by stock people who were trained for routinely performing euthanasia at the slaughterhouse. Pigs were bred in the temperature-controlled room with appropriate dark–light cycles, fed with regular diet, and maintained under the care of Wens modern hoggery (Taicang, Jiangsu) following their guidelines. Abattoir-derived porcine ovaries were transported to the laboratory within 2 h in the sterile physiological saline (0.9% NaCl) containing 500 IU/ml of both penicillin and streptomycin, maintained at 30–35 °C in a thermos bottle after slaughter. Soon afterwards ovaries were washed twice with sterile phosphate-buffered saline (PBS), the COCs (cumulus-oocyte complexes) were subsequently aspirated from medium-sized follicles (3–6 mm in diameter) using a 20-gauge needle attached to a 20-ml disposable syringe. COCs surrounded by a compact cumulus mass with evenly granulated cytoplasm were washed three times with maturation medium, separated from the cellular debris, and then transferred to the maturation medium. The basic maturation medium was improved TCM-199 supplemented with 75 μg/ml of penicillin, 50 μg/ml of streptomycin, 0.5 μg/ml of LH, 0.5 μg/ml of FSH, 10 ng/ml of epidermal growth factor (mouse EGF; Sigma) and 0.57 mM cysteine (Sigma). To prepare mature oocyte in vitro, a group of 80 COCs was transferred to 500 μl of maturation medium and then covered with 200 μl paraffin oil to culture at 38.5 °C in a humidified atmosphere of 5% CO_2_.

### Immunofluorescent and confocal microscopy

Denuded oocytes (DOs) were washed in PBS, and then fixed in 4% paraformaldehyde in PBS for 1 h at room temperature. Oocytes were washed 3 times in PBS, and then rehydrated and transferred to the permeabilization solution (1% Triton X-100, 20 mM HEPES, PH 7.4, 3 mM MgCl_2_, 50 mM NaCl, 300 mM sucrose, 0.02% NaN_3_ in PBS) for 8–12 h. After blocking with 3% BSA for 1 h at room temperature, oocytes were incubated with anti-α-tubulin-FITC antibody (1:200), anti-acetylated tubulin antibody (1:100), anti-phalloidin-FITC antibody (1:200) or rabbit polyclonal anti-mouse ovastacin antibody (1:100) at 4 °C overnight, followed by incubation with an appropriate secondary antibody for 1 h and counterstaining of PI (Propidium Iodide) for 10 min at room temperature. MitoTracker Red CMXRos (ThermoFisher, USA) was used to label the mitochondria. Finally, oocytes were mounted on glass slides and observed under a laser-scanning confocal fluorescent microscope (Zeiss LSM 700 META confocal system).

For measurement of fluorescence intensity, the signals from both control and treatment oocytes were acquired by performing the same immunostaining procedure and setting up the same parameters of confocal microscope. The average fluorescence intensity per unit area within the region of interest (ROI) was applied to quantify the fluorescence of each oocyte images. Fluorescence intensity was randomly measured by plot profiling using ImageJ software (NIH, USA).

### Western blotting analysis

A total of 100 porcine oocytes was collected and lysed in 4× NuPAGE™ LDS sample buffer (ThermoFisher, USA) containing protease inhibitor, and then separated on 10% Bis–Tris precast gels and transferred onto polyvinylidene difluoride (PVDF) membranes. The blots were blocked in Tris buffered saline Tween 20 (TBST) containing 5% low fat dry milk for 1 h at room temperature and then incubated with anti-acetylated tubulin antibody (1:1000) or anti-Gapdh (1:5000) antibody overnight at 4 °C. After washing in TBST, the blots were incubated with horseradish peroxidase (HRP)-conjugated secondary antibodies for 1 h at room temperature. Chemiluminescence was detected with ECL Plus (GE Healthcare, USA) and protein bands were visualized by Tanon-3900 (Tanon, China).

### Statistical analysis

All percentages from at least three repeated experiments were expressed as mean ± SEM, and the number of oocytes observed was labeled in parenthesesas (n). Data were analyzed by paired-samples *t* test, which was provided by GraphPad Prism5 statistical software. The level of significance was accepted as *p *<* 0.05*.

## Results

### Eg5 is essential for porcine oocyte meiotic maturation

**Fig. 1:**
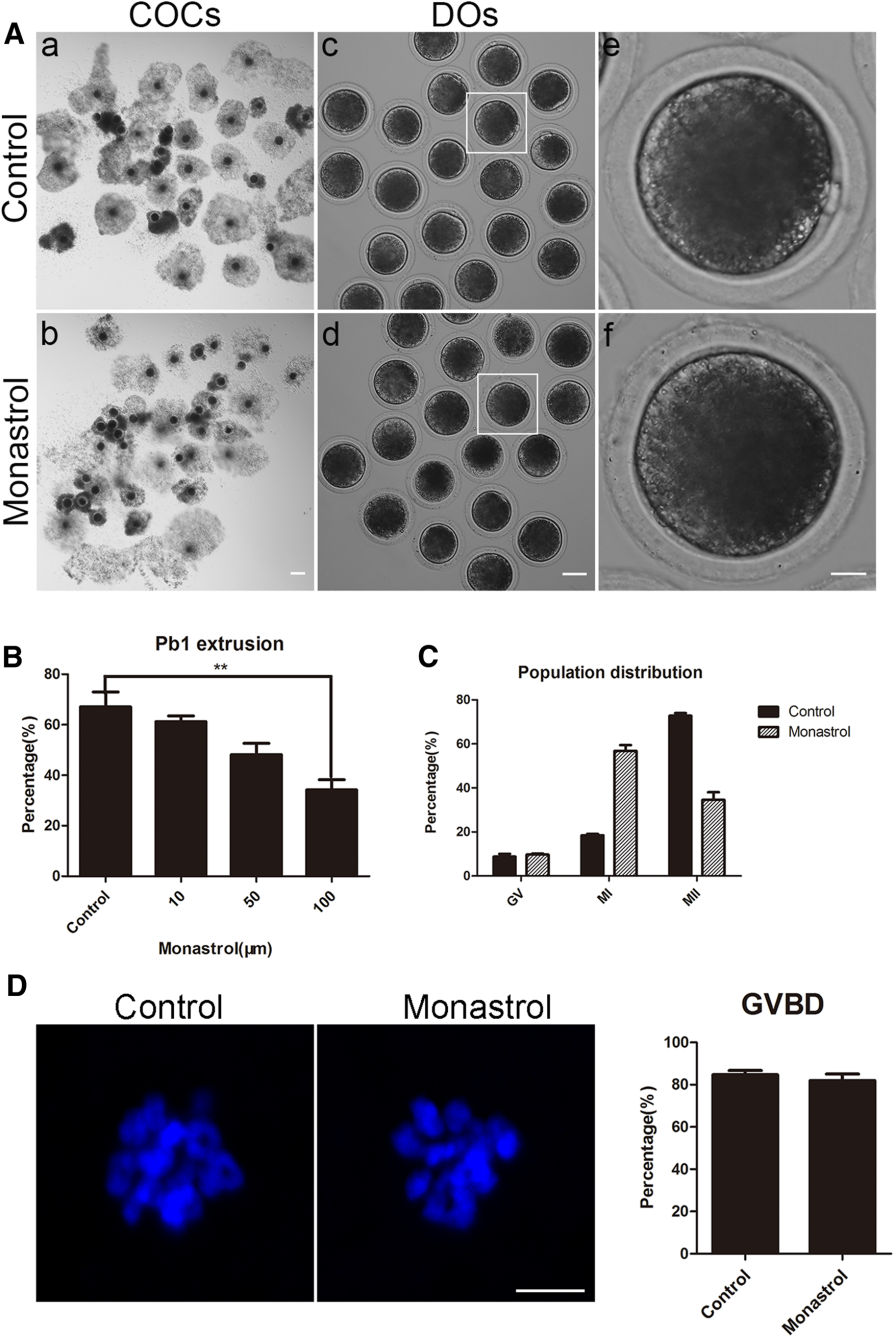
Effect of Eg5 inhibition on the porcine oocyte maturation. **A** Representative images of control and Eg5-inhibited oocytes cultured in vitro for 44 h. Cumulus cell expansion of COCs and polar body extrusion of DOs were imaged by confocal microscope. Scale bar, 150 μm (**a**, **b**); 80 μm (**c**, **d**); 20 μm (**e**, **f**). **B** The rates of polar body extrusion were recorded in control and different concentrations (10, 50 and 100 μM) of monastrol-treated oocytes. **C** The percentage of control and Eg5-inhibited oocytes in GV, M I or M II stage after in vitro maturation. **D** Oocytes that underwent GVBD were recorded in control and monastrol-treated groups. Scale bar, 5 μm. Data of **B**–**D** were presented as mean percentage (mean ± SEM) of at least three independent experiments. **p < 0.01

To examine the function of Eg5 during porcine oocyte maturation, a cell-permeable and specific inhibitor of Eg5, monastrol, was supplemented to the culture medium. As shown in Fig. 1, inhibition of Eg5 with monastrol treatment apparently led to the porcine oocyte meiotic failure by exhibiting the poor expansion of cumulus cells surrounding COCs and less matured M II eggs with first polar bodies (Fig. 1a). The quantification analysis showed that treatment with different concentrations of monastrol (10, 50 and 100 μM) resulted in the decreased rates of polar body extrusion (PBE) in varying degree in oocytes cultured for 44 h in vitro and supplementation with 100 μM inhibitor had a significant reduction compared to controls (control: 67.1 ± 5.9%, n = 96; 10 μM: 61.2 ± 2.3%, n = 93; 50 μM: 48.2 ± 4.5%, n = 106; 100 μM: 34.2 ± 4.1%, n = 99, *p *< 0.01; Fig. 1b). Thus, 100 μM monastrol was used for subsequent studies. We also counterstained the oocytes that did not extrude polar bodies with Hoechst and found that most of them were arrested at M I stage (Fig. 1c). In the meantime, the statistical result revealed that GVBD was not affected after monastrol treatment (81.9 ± 3.2%, n = 50 vs 84.7 ± 2.0%, n = 46 control; Fig. 1d). Taken together, these observations suggest that Eg5 is required for normal porcine oocyte meiotic progression.

### Inhibition of Eg5 perturbs the spindle assembly and chromosome alignment in porcine oocytes

**Fig. 2:**
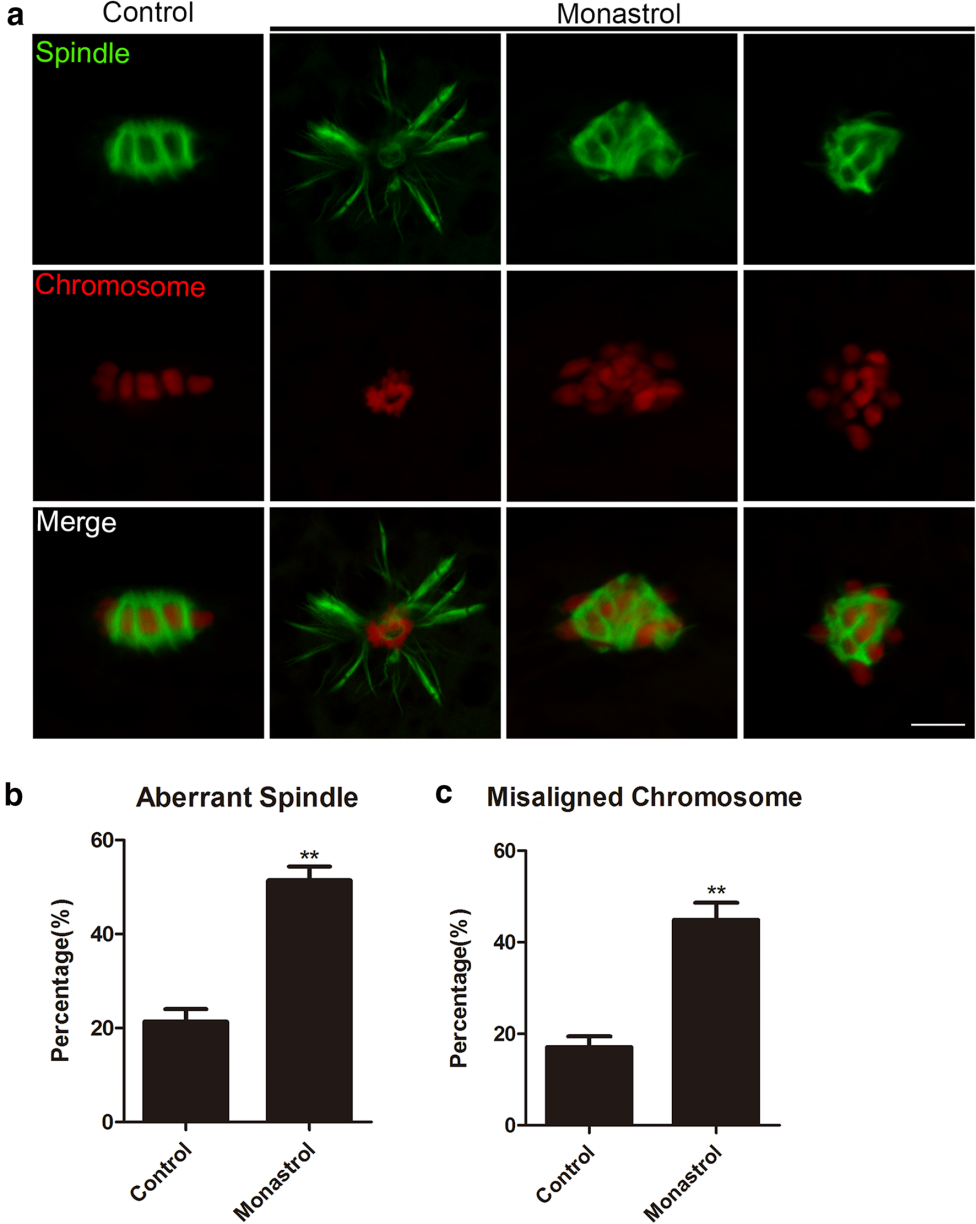
Effect of Eg5 inhibition on the spindle assembly and chromosome alignment in porcine oocytes. **a** Representative images of spindle morphologies and chromosome alignment in control and Eg5-inhibited oocytes. Oocytes were immnunostained with anti-α-tubulin-FITC antibody to visualize the spindles and counterstained with Hoechst to visualize the chromosomes. Scale bar, 5 μm. **b** The rate of aberrant spindles was recorded in control and Eg5-inhibited oocytes. **c** The rate of misaligned chromosomes was recorded in control and Eg5-inhibited oocytes. Data in **b**, **c** were presented as mean percentage (mean ± SEM) of at least three independent experiments. **p < 0.01

To test whether the meiotic failure present in Eg5-inhibited oocytes is caused by the spindle/chromosome abnormalities, M I oocytes cultured 28 h in vitro were immunostained with anti-α-tubulin-FITC antibody to observe the spindle organization and counterstained with PI (Propidium Iodide) to visualize the chromosome alignment. The immunofluorescence results revealed that most of control oocytes displayed a typical barrel-shape spindle morphology with a well-aligned chromosome on the equatorial plate (21.4 ± 2.6%, n = 49, spindle; 17.1 ± 2.3%, n = 49, chromosome; Fig. 2a–c). By contrast, Eg5-inhibited oocytes had a great number of abnormal spindles with misaligned chromosomes (51.4 ± 3.0%, n = 47, *p *< 0.01, spindle; 44.9 ± 3.8%, n = 47, *p *< 0.01, chromosome; Fig. 2a–c).

### Inhibition of Eg5 decreases the acetylation level of α-tubulin in porcine oocytes

**Fig. 3:**
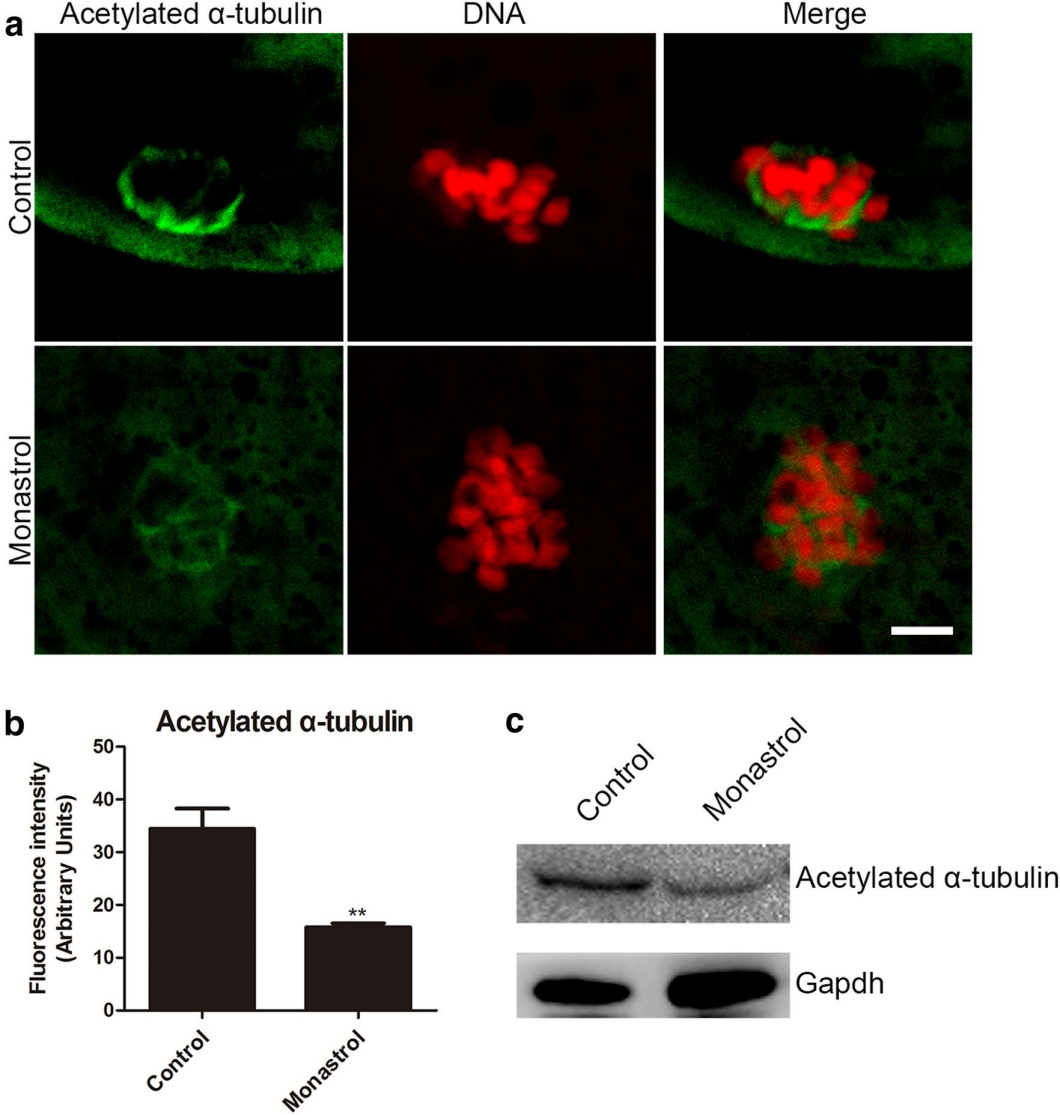
Effect of Eg5 inhibition on the acetylation level of α-tubulin in porcine oocytes. **a** Representative images of acetylated α-tubulin in control and Eg5-inhibited oocytes. Oocytes were immnunostained with anti-acetyl-α-tubulin (Lys-40) antibody to assess the acetylation level of α-tubulin. Scale bar, 5 μm. **b** Quantitative analysis of the fluorescence intensity of acetylated α-tubulin in control and Eg5-inhibited oocytes. Data were presented as mean percentage (mean ± SEM) of at least three independent experiments. **p < 0.01. **c** The acetylation levels of α-tubulin in control and Eg5-inhibited oocytes were examined by western blotting. The blots were probed with anti-acetyl-α-tubulin (Lys-40) antibody and anti-Gapdh antibody, respectively

The defects of spindle assembly prompted us to test the microtubule dynamics in Eg5-inhibited oocytes. Because previous reports have documented that acetylation level of α-tubulin is an indicator of the stabilized microtubules in both mitotic and meiotic cell [28–31], we therefore tested the microtubule stability in Eg5-inhibited oocytes at M I stage. We found that Eg5 inhibition considerably impaired the signals of acetylated α-tubulin in porcine oocytes, and the fluorescence intensity showed a remarkable decrease in the treatment group compared to the controls (15.8 ± 0.7, n = 37 vs 34.4 ± 3.8, n = 41, control, *p *< 0.01; Fig. 3a, b), which was further confirmed by the western blotting analysis (Fig. 3c). Thus, the hypoacetylation of α-tubulin indicates that microtubules are less stable in the Eg5-inhibited oocytes, which hence compromises the microtubule stability and spindle assembly.

### Inhibition of Eg5 disrupts the structure of actin filaments in porcine oocytes

**Fig. 4:**
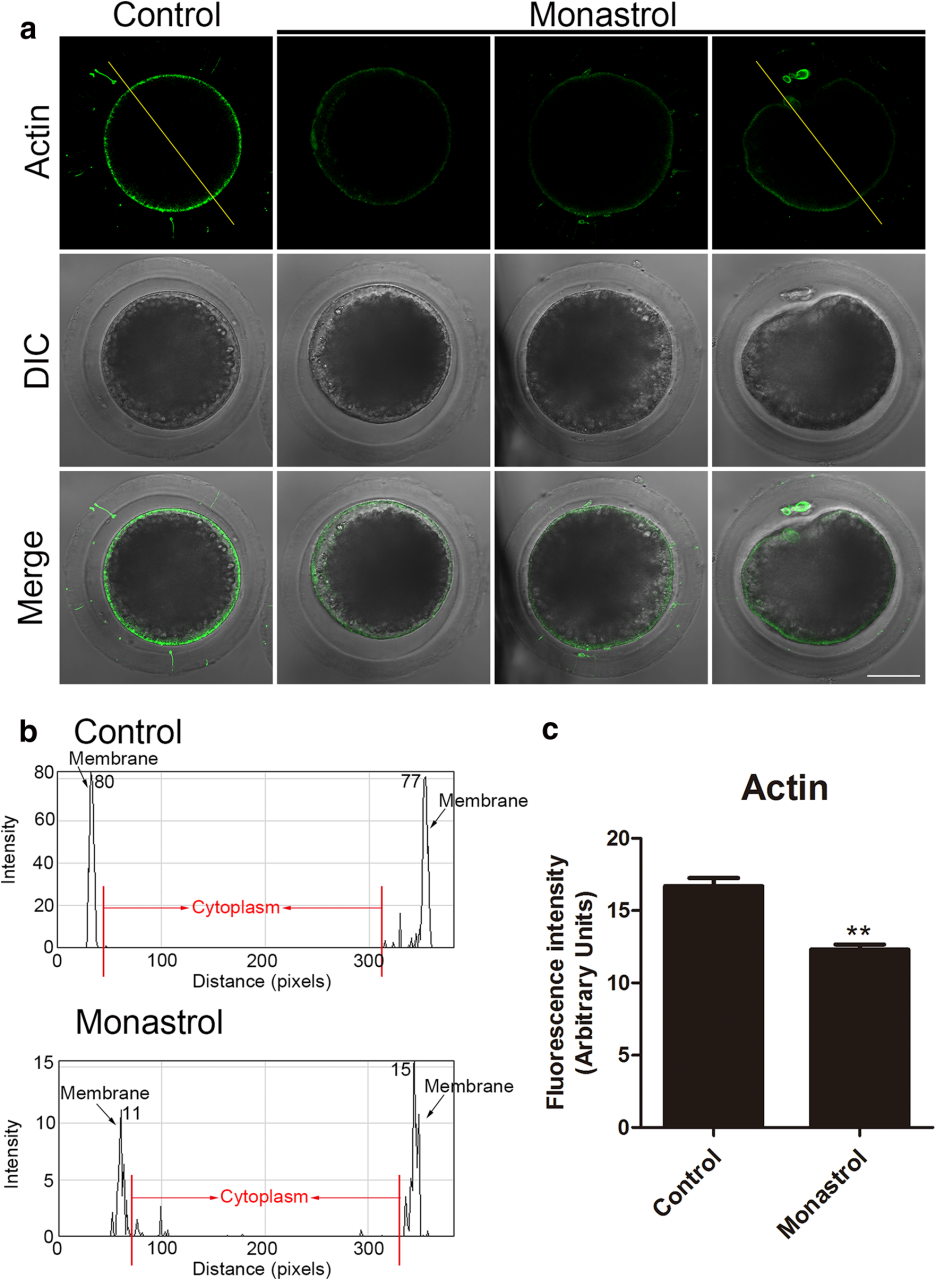
Effect of Eg5 inhibition on the actin dynamics in porcine oocytes. **a** Representative images of actin distribution in control and Eg5-inhibited oocytes. Oocytes were immnunostained with anti-phalloidin-FITC antibody to visualize the actin filaments. Scale bar, 40 μm. **b** Right graphs show fluorescence intensity profiling of phalloidin in oocytes. Lines were drawn through the oocytes, and pixel intensities were quantified along the lines. **c** The fluorescence intensity of actin signals was measured in control and Eg5-inhibited oocytes. Data were presented as mean percentage (mean ± SEM) of at least three independent experiments. **p < 0.01

Dynamic actin polymerization drives meiotic spindle migration, spindle positioning, intracellular transportation and cytokinesis in cells [32]. To further examine whether Eg5 promotes the oocyte maturation by affecting the actin cytoskeleton, phalloidin was used as a probe to stain the F-actin in M I oocytes cultured 28 h in vitro. In control oocytes, the robust signals of actin were distributed evenly in the plasma membrane (Fig. 4a). However, Eg5-inhibited oocytes exhibited a discontinuous distribution of actin filaments with weak signals (Fig. 4a), which was confirmed by fluorescence plot profiling (Fig. 4b). The quantitative measurement of fluorescence intensity showed that actin signals were significantly decreased when Eg5 was inhibited compared to controls (12.3 ± 0.3, n = 32 vs 16.7 ± 0.6, n = 36, control, *p *<0.01; Fig. 4c), suggesting that Eg5 is involved in the actin dynamics to ensure the meiotic progression.

### Inhibition of Eg5 impairs the mitochondrial integrity in porcine oocytes

**Fig. 5:**
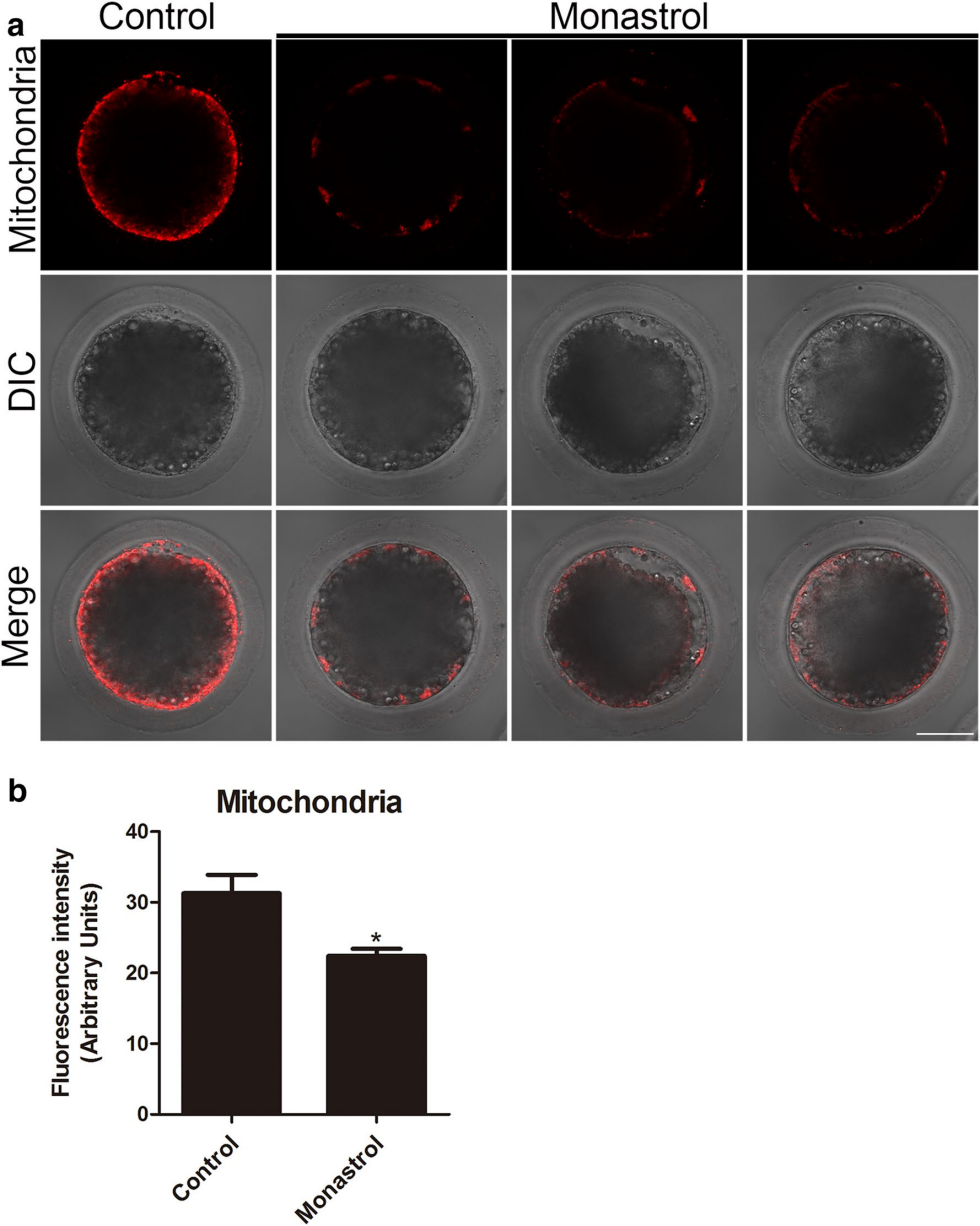
Effect of Eg5 on the mitochondrial integrity in porcine oocytes. **a** Representative images of mitochondrion distribution in control and Eg5-inhibited oocytes. Oocytes were labeled with MitoTrackerR to visualize the mitochondria. Scale bar, 40 μm. **b** The fluorescence intensity of mitochondrion signals was measured in control and Eg5-inhibited oocytes. Data were presented as mean percentage (mean ± SEM) of at least three independent experiments. *p < 0.05

Because mitochondrial integrity is one of the critical indicators of high-quality oocytes, we then assessed its changes in inhibition of Eg5 in M II oocytes cultured 44 h in vitro. As shown in Fig. 5, mitochondria accumulated around the lipid droplets in most of control porcine oocytes, but displayed the discontinuous localization with faded signals in Eg5-inhibited oocytes (Fig. 5a). By fluorescence measurement, we found that the signal intensity of mitochondria has a remarkable reduction in Eg5-inhibited oocytes compared to the controls (22.4 ± 1.0, n = 43 vs 31.3 ± 2.6, n = 45, control, *p *<0.05; Fig. 5b), suggesting that mitochondrial integrity could not be maintained when Eg5 activity is impeded.

### Eg5 is required for the proper distribution of CGs and ovastacin in porcine oocytes

**Fig. 6:**
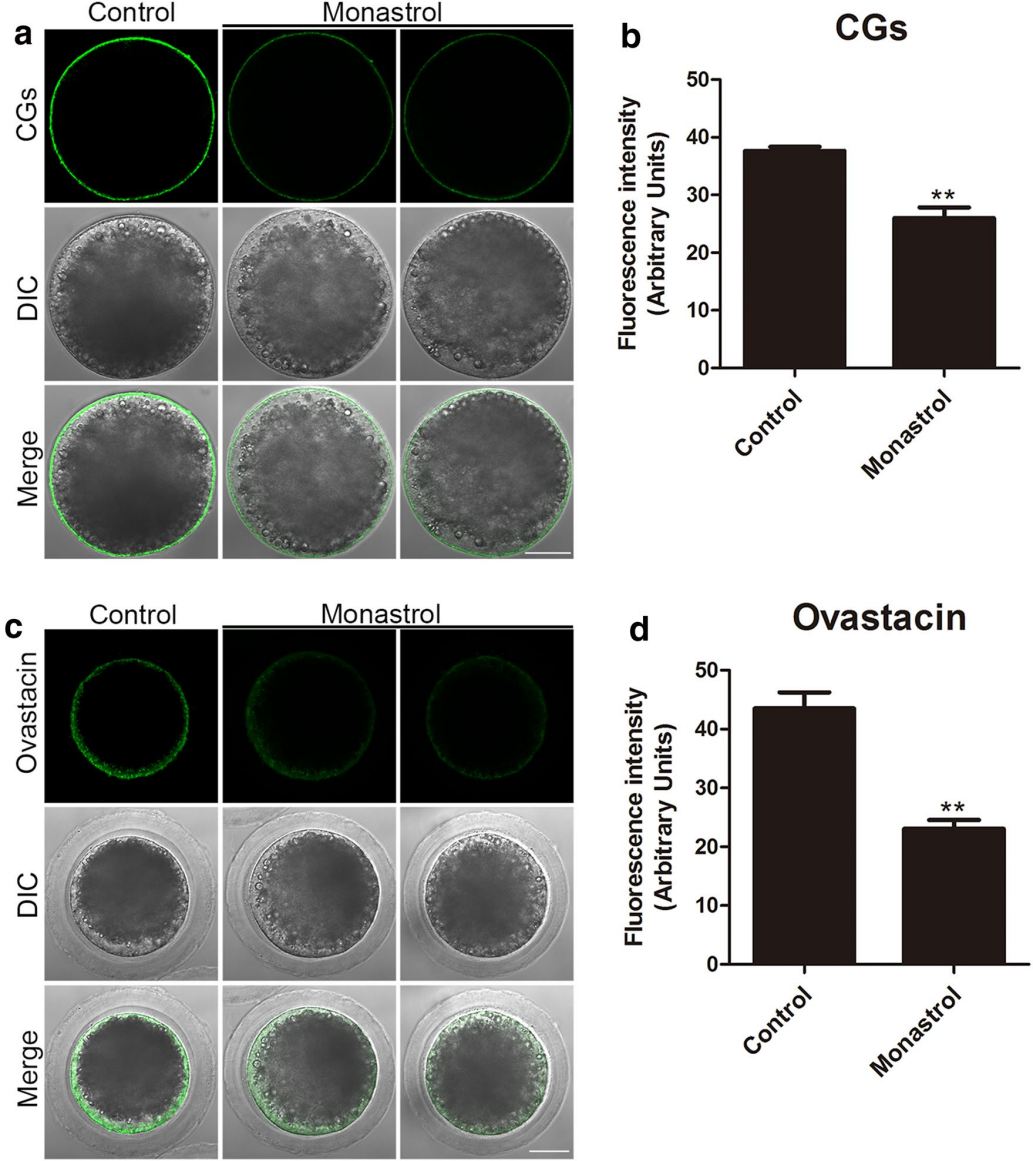
Effect of Eg5 inhibition on the distribution of cortical granules and ovastacin in porcine oocytes. **a** Representative images of cortical granule localization in control and Eg5-inhibited oocytes. Oocytes were immnunostained with LCA-FITC to visualize the cortical granules. Scale bar, 40 μm. **b** The fluorescence intensity of cortical granules was measured in control and Eg5-inhibited oocytes. **c** Representative images of ovastacin localization in control and Eg5-inhibited oocytes. Ovastacin was immunostained with rabbit polyclonal anti-human ovastacin antibody and imaged by confocal microscopy. Scale bar, 40 μm. **d** The fluorescence intensity of ovastacin was measured in control and Eg5-inhibited oocytes. Data in **b** and **d** were presented as mean percentage (mean ± SEM) of at least three independent experiments. **p < 0.01

CGs (cortical granules) are Golgi apparatus-derived and oocyte-specific vesicles which are well known for their function in the block to polyspermy. Normal distribution of CGs is usually considered as one of the most important indicators of oocyte cytoplasmic maturation. Thus, the localization of CGs was examined with their marker LCA-FITC in M II oocytes cultured 44 h in vitro and imaged by confocal microscope. The immunostaining result revealed that Eg5 inhibition considerably compromised the proper localization of CGs in the oocyte cortex and the fluorescence intensity of CGs had a pronounced reduction in comparison with the controls (26.0 ± 1.8, n = 52 vs 37.6 ± 0.7, *p *< 0.01, n = 49; Fig. 6a, b). Additionally, ovastacin is the component of CGs which is responsible for the post-fertilization cleavage of sperm recognizing site ZP2 to block sperm binding and polyspermy. We also examined its distribution after inhibition of Eg5. Mis-localization of ovastacin was observed in Eg5-inhibited oocytes by showing the loss of continuous distribution in oocyte cortical region and lower signals of fluorescence intensity than those in control oocytes (23.1 ± 1.5, n = 39 vs 43.5 ± 2.8, n = 44, *p *<0.01; Fig. 6c, d), implying that fertilization ability might be impaired in Eg5-inhibited oocytes.

## Discussion

Kinesins are a family of molecular motors that travel unidirectionally along microtubule tracks to fulfil their numerous roles in intracellular transport and other biological events [33]. Among these motor proteins, of particular interest is Eg5, a member of kinesin-5 motors which are highly conserved throughout the eukaryote lineage [34]. It has found that Eg5 can tether microtubule plus-ends to participate in spindle formation in mitosis and sister chromatid separation in meiosis [11, 25]. To investigate the potential roles of Eg5 during oocyte meiotic maturation, we selected porcine oocytes as a model because they are physiologically and developmentally more similar to human oocytes than mouse oocytes. During the procedure of human assisted reproductive technology, GV oocytes are retrieved from patients and then in vitro cultured to MII eggs for in vitro fertilization or intracytoplasmic sperm injection. Nevertheless, many oocytes would suffer from the maturation arrest during this process, and the underlying genetic cause is less known. Until recently, a genetic study reported that *TUBB8* mutations have dominant-negative effects that disrupt microtubule behavior and oocyte meiotic spindle assembly and maturation, causing female infertility [35]. However, more molecules that are involved in this process needs to be identified. Thus, our present study is aimed to mimics the in vitro oocyte maturation procedure in human clinics to study the role of Eg5, which would provide some implications for the cause of oocyte maturation arrest and human reproductive research.

We first of all examined the impact of the inhibitor of Eg5, monastrol, on the porcine oocyte meiotic maturation. With the treatment of increasing doses of the inhibitor, the oocytes exhibited a significantly decreased rate of polar body extrusion with poor expansion of cumulus cells, suggesting that inhibition of Eg5 perturbs the meiotic progression. Previous studies by us and others have shown that oocyte meiotic arrest is largely caused by the defective spindle assembly and chromosome alignment [31, 36, 37]. Additionally, Eg5 has been found to sort and push microtubules apart to drive the separation of spindle poles [12], and loss of Eg5 leads to cell cycle arrest and defective centrosome separation resulting in the development of monopolar spindles [38]. Consistently, our findings showed that spindle assembly and chromosome alignment were severely disrupted in Eg5-inhibited porcine oocytes. Different from the study in mouse oocytes that monastrol treatment also perturbs the event of GVBD besides the metaphase I arrest caused by the occurrence of monoastral spindles and chromosome displacement from the metaphase plate [25], our findings revealed that Eg5 inhibition had no effect on the GVBD. Furthermore, in Xenopus oocytes, Eg5 is localized both along the microtubules between the spindle poles and chromosomes of meiotic spindles [39]. Although Eg5-inhibited oocytes exhibit the monopolar spindles, they undergo the monopolar anaphase on time without additional intervention, suggesting that the meiotic progression is not affected by the Eg5 inhibition [40].

The involvement of Eg5 in the spindle organization further prompted us to assess its possible function in microtubule dynamics. Tubulin acetylation that occurs on Lys-40 of the α-tubulin subunit is found in both somatic cells and mouse oocytes as an indicator of stabilized microtubules [30, 31, 41, 42]. We then tested the acetylation level of α-tubulin as the indicator of microtubule stability in Eg5-inhibited oocytes. Our observations indicated that Eg5 inhibition greatly decreased the level of acetylated α-tubulin in porcine oocytes, suggesting that the loss of microtubule stability might be one of the leading causes resulting in the impaired spindle organization in Eg5-inhibited oocytes.

Actin filament is another essential component of cytoskeleton which functions in the intracellular transport, spindle positioning and cell cycle progression in both mitosis and meiosis [32]. We next determined the distribution of actin filament caused by Eg5 inhibition and validated that the impaired actin polymerization and distribution might be another important reason leading to the porcine oocyte meiotic failure.

Since mitochondrion is one of the most important organelles representing the primary source of ATP for normal oocyte and early embryo development [43], the distribution of mitochondria was detected in Eg5-inhibited oocytes. We found that the mitochondrial integrity displayed by the signal intensity was considerably diminished, indicating that Eg5 does impair the functionality of the mitochondria during porcine oocyte meiotic maturation.

Mammalian cortical granules are oocyte-specific and membrane-bound vesicles that accumulate during oogenesis and translocate to the subcortical region of fully grown eggs [7]. The proper localization of cortical granule under the oocyte cortex is usually regarded as a symbol of oocyte cytoplasmic maturation. We therefore ask whether inhibition of Eg5 would compromise the cortical granule dynamics. Our data revealed that the amount of cortical granules was remarkably reduced in Eg5 inhibited oocytes, indicating that cytoplasmic maturation is disrupted in this condition. Moreover, we tested the distribution of ovastacin, a pioneer cortical granule component that is responsible for post-fertilization cleavage of sperm binding site ZP2 for definitive block to polyspermy [44, 45]. Consistent with the defective dynamics of cortical granules, the amount of ovastacin was decreased in Eg5-inhibited oocytes, implying that ovastacin might be precociously released to the extracellular space to cleave ZP2 in the zona pellucida surrounding unfertilized eggs so as to cause the sperm binding failure.

## Conclusions

In summary, we demonstrate that Eg5 exerts an important function in the porcine oocyte meiotic maturation via maintaining the meiotic organelle arrangement, including spindle assembly, chromosome alignment, actin dynamics, mitochondrial integrity and cortical granule distribution. In addition, our findings extend our understanding of the molecular basis underlying the oocyte meiotic arrest that frequently occurs in human clinics.

## Abbreviations

PBE: polar body extrusion
M I: metaphase I
M II: metaphase II
SAC: spindle assembly checkpoint
DOs: denuded oocytes
COCs: cumulus-oocyte complexes
CGs: cortical granules
